# The impact of education and occupation on cognitive impairment: a cross-sectional study in China

**DOI:** 10.3389/fnagi.2024.1435626

**Published:** 2024-07-11

**Authors:** Tangsheng Zhong, Shiyuan Li, Peiqi Liu, Yonghong Wang, Li Chen

**Affiliations:** ^1^School of Nursing, Jilin University, Changchun, China; ^2^First Hospital of Jilin University, Changchun, China

**Keywords:** cognitive reserve, education, occupation, cognitive activity, cognitive impairment

## Abstract

**Background and objectives:**

Education, occupation, and cognitive activity are key indicators of cognitive reserve and are thought to influence cognitive impairment. However, the individual and combined impacts of these factors are not fully understood. This study aims to investigate the roles of education and occupation in cognitive impairment while controlling for brain reserve and cognitive activity.

**Methods:**

This cross-sectional study involved 369 participants aged 50 years or older from urban outpatient clinics in Jilin Province, China. Cognitive impairment was assessed using neuropsychological scales and brain imaging. Cognitive activity was evaluated with the Cognitive Reserve Scale (CRS). Covariance analysis and logistic regression models were used to analyze the associations, adjusting for age, sex, education, and occupation.

**Results:**

Higher education was significantly associated with a lower risk of cognitive impairment (*p* < 0.001), regardless of occupation. In contrast, occupational complexity and cognitive activity did not show a significant relationship with cognitive impairment (*p* > 0.05).

**Conclusion:**

Education, rather than occupation or cognitive activities, is a significant predictor of cognitive impairment, highlighting the importance of educational attainment in cognitive health.

## 1 Introduction

Cognitive reserve (CR) has become a cornerstone in understanding the resilience observed in individuals who exhibit minimal cognitive decline despite significant brain pathologies such as Alzheimer’s disease and other neurodegenerative conditions (Cavedo et al., 2012; Staff, 2012). The concept of CR explains the disparity between the extent of neuropathology and its clinical manifestations. CR is thought to be facilitated by lifelong intellectual engagement, encompassing formal education, challenging occupations, and leisure activities (Stern, 2012; Lövdén et al., 2020). This study aims to investigate the specific roles of education, occupation, and cognitive activity in contributing to CR and their individual and combined impacts on cognitive impairment. We hypothesize that higher education and intellectually demanding occupations will be associated with higher CR and better cognitive outcomes.

A newly published white paper used CR to refer to an adaptive force that helps explain differences in sensitivity among individuals when coping with aging or lesions (Stern et al., 2020). CR is defined within this study as the brain’s capacity to resist neurodegenerative damage due to its cognitive and neural flexibility. This section aims to expand on this operational definition by exploring the mechanisms through which intellectual engagements–such as education, challenging occupations, and leisure activities–contribute to CR. These activities are believed to enhance neural efficiency and cognitive flexibility, thereby allowing individuals to better manage the cognitive demands imposed by brain pathology.

Building on the definition of CR, our study particularly emphasizes the role of education and occupation. We posit that higher educational attainment and engagement in intellectually demanding occupations bolster cognitive reserve by enhancing synaptic density, network connectivity, and overall neural plasticity (Li et al., 2021; Ekdahl et al., 2023). This segment delves into how these components interact with lifelong cognitive activities to mitigate the effects of neurodegenerative lesions, providing a more detailed examination of the pathways through which education and occupational complexity influence cognitive health.

Research into CR has significantly advanced; however, critical gaps remain regarding the quantification of how educational and occupational engagements individually and collectively impact cognitive decline (Sherman-Wilkins and Thierry, 2019; Corbo et al., 2023). Our study seeks to address these gaps by employing advanced statistical models to quantify the relationship between CR components and cognitive outcomes. These models aim to refine our understanding and prediction of cognitive decline, paving the way for more targeted preventive and therapeutic interventions. Specifically, we introduce predictive modeling as a methodological approach to assess how educational attainment and occupational complexity contribute to cognitive reserve in aging populations.

This study proposes to test two primary hypotheses: (1) higher levels of educational attainment and (2) more complex occupational tasks are independently and synergistically associated with greater cognitive reserve. Furthermore, this cognitive reserve is hypothesized to influence cognitive performance among elderly subjects. We assess cognitive performance using standard neuropsychological assessments rather than direct measures of brain pathology. This approach allows us to infer the protective effects of cognitive reserve against cognitive decline without directly measuring neurodegenerative markers, providing insights into how lifelong educational and occupational engagements contribute to resilience against cognitive impairment.

## 2 Methods

### 2.1 Study participants and design

This cross-sectional study recruited participants from urban outpatient clinics of Jilin Province, China, reflecting diverse socio-economic conditions. Individuals undergoing medical examination were recruited from the largest regional medical center in the city. The inclusion criteria were as follows: aged 50 years or older, native Chinese speaker (able to communicate without barriers), and a Mini-Mental State Examination (MMSE) score of>24 points (primary: >20 points; illiteracy: >17 points). Written informed consent was obtained from all participants. Recruitment began in October 2022 and concluded in March 2023. Neurologists interviewed all subjects, and any individuals with a history of psychiatric or central nervous system disorders, hearing loss, learning disabilities, or any other conditions that could impact their performance on the neuropsychological test battery were excluded. Interviewers were trained to recognize when information on one questionnaire contradicts another, and we excluded participants with any signs of inconsistency found during the interview. This study was performed per the ethical standards in the 1964 Declaration of Helsinki, and the Ethics Committee of the School of Nursing, Jilin University approved the study and registered it with the China Clinical Registry (ChiCTR2200055112). A total of 378 participants were recruited, of which nine did not meet the inclusion and exclusion criteria or withdrew from the survey.

### 2.2 Cognitive reserve assessment

Cognitive Reserve was quantitatively assessed using the Cognitive Reserve Scale (CRS), a tool specifically designed to evaluate lifetime cognitive activities across diverse domains (Leon et al., 2011; León et al., 2014; Roldán-Tapia et al., 2017; León et al., 2018). This scale systematically gathers data on education, work complexity, and leisure activities, producing a composite score that reflects each participant’s overall cognitive reserve.

The CRS is structured into four categories: daily activities, training, hobbies, and social life, with items rated on a 5-point Likert scale ranging from 0 (never) to 4 (three or more times per week). This method highlights the cumulative cognitive capacity of individuals, focusing on sustained cognitive engagement rather than transient cognitive activities. It addresses three critical life stages: youth (18–35 years), middle age (36–64 years), and old age (≥65 years). Participants complete the subscale corresponding to their current age stage. Each item’s score is summed to derive a total CRS score, which can range from 0 to 96. Higher scores indicate more frequent engagement in cognitive reserve-building activities across the assessed life stages, reflecting higher levels of cognitive reserve.

### 2.3 Neuropsychological evaluation

To thoroughly assess the cognitive performance of participants, our study employed a comprehensive neuropsychological test battery that included well-established cognitive assessments alongside additional tests aimed at covering a broad spectrum of cognitive functions. Key instruments used were the MMSE (Katzman et al., 1988) and the Montreal Cognitive Assessment (MoCA) (Lu et al., 2011), both highly regarded for their utility in identifying cognitive impairments across various cognitive domains. Importantly, in order to ensure accuracy and relevance to our demographic, the raw scores for both the MMSE and MoCA were adjusted for age and educational level based on Chinese normative data. This adjustment is crucial as it accounts for the significant impact that these demographic factors can have on cognitive testing outcomes, thereby allowing for a more precise evaluation of cognitive health relative to the normative standards of the Chinese population.

Furthermore, the study incorporated the Activities of Daily Living (ADL) scale to evaluate functional capabilities (Lawton and Brody, 1969; Graf, 2008), the Auditory Verbal Learning Test (AVLT) (Dong et al., 2023) for memory and learning abilities, and the Memory and Executive Screening (MES) test (Guo et al., 2012) to probe executive functions and memory recall. These assessments are especially sensitive to cognitive changes potentially related to varying levels of cognitive reserve.

Additionally, our neuropsychological evaluation featured tasks aimed at assessing verbal fluency, abstract reasoning, and complex problem-solving capabilities, such as the Animal Naming Test (ANT) (Huang et al., 2023) and the Clinical Dementia Rating (CDR) scale (Morris, 1993), which assesses six cognitive and functional domains including memory, orientation, judgment and problem-solving, community affairs, home and hobbies, and personal care (Howell et al., 2022). This robust approach aids in effectively detecting cognitive impairments, defining cognitive impairment as a CDR score of 0.5 or greater.

These tests, conducted under standardized conditions, were designed to collect detailed data on the cognitive health of participants, with results thoroughly documented and analyzed. By correlating cognitive performance with Cognitive Reserve Scale scores, our research aims to explore how cognitive reserve levels influence individuals’ vulnerability or resilience to cognitive impairments.

This comprehensive evaluation serves to elucidate the intricate relationship between cognitive reserve and cognitive performance, offering deeper insights into cognitive reserve’s potential to protect against cognitive decline.

### 2.4 Measurement of brain reserve

Brain reserve refers to the brain’s ability to tolerate pathology without exhibiting clinical symptoms, a concept that is supported by varying individual resilience to similar levels of brain pathology (Satz, 1993; Hachinski and Avan, 2022). Recent research underscores the importance of brain reserve in mediating the effects of neurological changes, particularly in the context of aging and neurodegenerative diseases (Chen et al., 2020; Stern et al., 2020). This variability can be explored through proxy measures when direct neuroimaging is not feasible.

While advanced neuroimaging techniques have offered deep insights into brain reserve by measuring aspects such as cortical thickness and white matter integrity, not all studies have the resources to employ such methods. In this context, simpler, more accessible proxy measures like head circumference have been used as indicators of brain reserve (Chen et al., 2020; Yang et al., 2020). Head circumference is thought to correlate with brain volume, which is a critical component of brain reserve (Yang et al., 2020). Larger brain volumes are hypothesized to provide a greater buffer against the cognitive impairments resulting from brain pathologies, such as those seen in Alzheimer’s disease.

This study utilizes head circumference as a proxy for brain reserve, drawing on methodologies that, while less direct than neuroimaging, still offer valuable insights into the structural capacities that might underpin cognitive resilience.

### 2.5 Statistical analysis

Data were analyzed using IBM SPSS Statistics 25 (IBM Corp, Armonk, NY, United States). Descriptive statistics were computed for all primary variables, reported as mean ± SD or median (Q1–Q3) for numerical data and N (%) for categorical data. To address the issue of unbalanced age groups, weights were applied in all analyses to adjust for disproportionate sample sizes across age categories. This adjustment ensures a more accurate representation and analysis of the data. Categorical variables were compared using chi-square tests, and continuous variables were assessed using unpaired t-tests.

Categorical variables were compared using chi-square tests, and continuous variables were assessed using unpaired t-tests to evaluate differences between participants with and without cognitive impairment in sociodemographic and clinical variables. Additionally, a variance inflation factor (VIF) analysis was conducted to check for multicollinearity among predictors before running the regression models.

The relationship between cognitive reserve (quantified by CRS total scores) and neuropsychological outcomes was examined using multiple regression models. These models included adjustments for age, sex, and educational level, as well as other potential confounders identified during the multicollinearity analysis. Additionally, a factorial ANOVA was conducted to explore the interactions between education and occupation on CRS scores, with age as a covariate. The significance threshold for all tests was set at *α* < 0.05.

## 3 Results

### 3.1 Demographic and clinical characteristics

The demographic and clinical characteristics of the 369 participants are summarized in Table 1. The average age of participants was 61.5 years (SD = 7.9). The cohort included 156 males (42.3%) and 213 females (57.7%). Significant differences were noted in the rates of cognitive impairment among participants, which varied by age, educational level, waistline, thigh circumference, and family history of stroke. Older adults and those with less education were more likely to exhibit signs of cognitive impairment, with statistical significance observed across these groups (*p* < 0.05).

**Table 1:** Characteristics of the sample according to the presence of cognitive impairment (*n* = 369).

Sociodemographic variable	All	Cognitive impairment		t/χ^2^-Value	*p*-Value
	(n = 369)	No(n = 240)	Yes(n = 129)		
Age (years), mean ± SD^*^	61.5 ± 7.9	60.0 ± 7.9	64.3 ± 7.2	−5.018	<0.001
Age group, *n* (%)^†^				15.369	<0.001
<65 years	251 (68.0)	180 (75.0)	71 (55.0)		
≥65 years	118 (32.0)	60 (50.8)	58 (49.2)		
Male sex, n(%)^†^				0.973	0.377
Male	156 (42.3)	97 (62.2)	59 (37.8)		
Female	213 (57.7)	143 (67.1)	70 (32.9)		
Education level, *n* (%)^†^				18.949	<0.001
Lower education	91 (24.7)	42 (46.2)	49 (53.8)		
Higher education	278 (75.3)	198 (71.2)	80 (28.8)		
Occupation, *n* (%)^†^				2.150	0.177
Physical	76 (20.6)	44 (57.9)	32 (42.1)		
Intellectual	293 (79.4)	196 (66.9)	97 (33.1)		
Cognitive activity total, mean ± SD^*^	42.6 ± 12.0	43.2 ± 11.5	41.5 ± 12.8	1.254	0.211
Cognitive activity stage, mean ± SD^*^					
Youth (score)	44.5 ± 12.9	45.4 ± 11.8	42.8 ± 14.6	1.770	0.078
Middle age (score)	41.4 ± 13.2	41.4 ± 13.1	41.4 ± 13.5	−0.029	0.977
Old age (score)	40.3 ± 13.0	41.2 ± 13.3	39.5 ± 12.8	0.699	0.486
Cognitive activity grade, *n* (%)^†^				0.829	0.676
Low	99 (26.8)	67 (67.7)	32 (32.3)		
Medium	171 (46.3)	112 (65.5)	59 (34.5)		
High	99 (26.8)	61 (61.6)	38 (38.4)		
Circumference of body parts (cm), mean ± SD^*^					
Head circumference	55.1 ± 2.5	55.0 ± 2.5	55.3 ± 2.4	−1.306	0.192
Height	164.4 ± 7.6	164.4 ± 7.9	164.4 ± 7.2	−0.022	0.982
Waistline	84.3 ± 11.0	82.8 ± 11.7	87.1 ± 9.1	−3.861	<0.001
Hipline	99.9 ± 7.7	100.0 ± 8.2	99.7 ± 6.7	0.374	0.709
Upper limb circumference	29.1 ± 3.9	28.8 ± 3.9	29.6 ± 3.7	−1.897	0.059
Thigh circumference	50.1 ± 6.8	49.5 ± 6.7	51.0 ± 6.9	−2.074	0.039
Calf circumference	35.6 ± 3.8	35.4 ± 4.0	36.0 ± 3.5	−1.654	0.099
Weight (kg), mean ± SD^*^	66.5 ± 11.5	66.6 ± 11.9	66.4 ± 10.8	0.167	0.867
Regular exercise habit, *n* (%)^†^				0.329	0.548
No	57 (15.4)	35 (61.4)	22 (38.6)		
Yes	312 (84.6)	205 (65.7)	107 (34.3)		
Social activities, *n* (%)^†^				1.351	0.521
Low	126 (34.1)	87 (69.0)	39 (31.0)		
Medium	170 (46.1)	107 (62.9)	63 (37.1)		
High	73 (19.8)	46 (63.0)	27 (37.0)		
Family history, *n* (%)^†^					
Dementia	52 (14.1)	36 (15.0)	16 (12.4)	0.467	0.534
Stroke	85 (23.0)	65 (27.1)	20 (15.5)	6.346	0.014
Hypertension	160 (43.4)	110 (45.8)	50 (38.8)	1.709	0.226
Diabetes	97 (26.3)	63 (26.3)	34 (26.4)	<0.001	0.982
Dyslipidemia	65 (17.6)	49 (20.4)	16 (12.4)	3.713	0.062
CHD	96 (26.0)	69 (28.7)	27 (20.9)	2.666	0.108
Cancer	68 (18.4)	51 (21.3)	17 (13.2)	3.636	0.067

*Unpaired *t* test. †Chi-squared test.

### 3.2 Distribution of cognitive impairment

**Figure 1:**
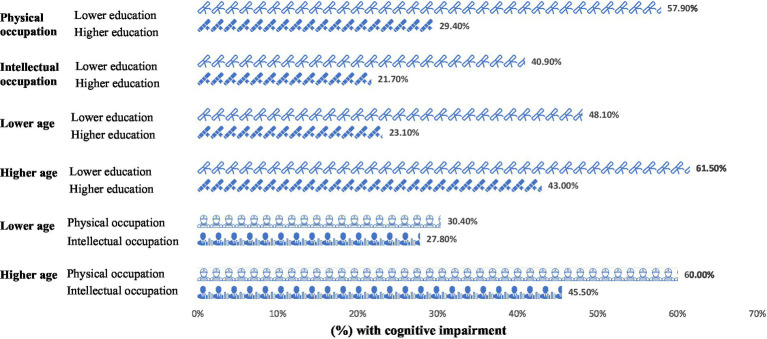
Proportion of cognitive impairment across different participant groups (*N* = 369). Lower education = years of education≤12 years; Higher education: university degree or above; Physical occupation: occupation predominated by physical strength; Intellectual occupation: occupation predominated by intellectual skills; Lower age: <65 years; Higher age: ≥65 years.

The analysis explored the distribution of cognitive impairment rates across various demographic and occupational groups within our study cohort. Figure 1 depicts this distribution, highlighting significant disparities in cognitive impairment rates based on age, education level, and type of occupation.

The findings demonstrate that cognitive impairment was most prevalent among older adults, those with a lower level of education, and individuals engaged in physically demanding occupations. Specifically, over 60% of participants in these groups exhibited cognitive impairment, underscoring the pronounced impact of socio-economic and demographic factors on cognitive health.

Statistical analysis revealed that individuals with lower educational attainment faced a higher risk of cognitive impairment, irrespective of their occupation being intellectually or physically oriented in Table 2. The rates of cognitive impairment were significantly higher in individuals with lower education levels working in physical roles (*p* < 0.001) and those in intellectual roles (*p* = 0.018), suggesting that education level plays a crucial role in mitigating cognitive decline, beyond the nature of occupational activities.

**Table 2:** Association of cognitive impairment with education level and occupation (*n* = 369).

Group	Subgroup	*n*	Cognitive impairment			*χ*2-Value	df	*p*-value
			No	Yes	%			
Physical occupation	Lower education	38	16	22	57.90%	12.115	1	0.001
	Higher education	255	180	75	29.40%			
Intellectual occupation	Lower education	53	26	27	40.90%	5.612	1	0.018
	Higher education	23	18	5	21.70%			
Lower age	Lower education	52	27	25	48.10%	12.663	1	<0.001
	Higher education	199	153	46	23.10%			
Higher age	Lower education	39	15	24	61.50%	3.576	1	0.059
	Higher education	79	45	34	43.00%			
Lower age	Physical occupation	46	32	14	30.40%	0.128	1	0.720
	Intellectual occupation	205	148	57	27.80%			
Higher age	Physical occupation	30	12	18	60.00%	1.894	1	0.169
	Intellectual occupation	88	48	40	45.50%			

df, Degrees of Freedom.

Intriguingly, among the lower age population, differences in education levels significantly influenced the likelihood of experiencing cognitive impairment, with those attaining higher education faring better (*p* < 0.001). However, in the elderly population, this protective effect of higher education appeared diminished, with no statistically significant differences in cognitive impairment rates observed across educational levels. This pattern suggests that while higher education can delay the onset of cognitive impairments during earlier stages of adulthood, its protective effects may wane as individuals age.

### 3.3 Cognitive reserve and neuropsychological outcomes

Multivariable regression analysis detailed in Table 3 demonstrates that higher CRS scores significantly enhance neuropsychological performance, particularly in the MoCA and MES tests. Before conducting the final analysis, the VIF was used to check for multicollinearity among predictors to ensure the validity of the results. After adjusting for age, gender, and education level, each incremental increase in CRS scores yielded a 0.027-point increase in MoCA scores [*p* = 0.045, 95% CI (0.001, 0.054)] and a 0.121-point increase in MES scores [*p* = 0.008, 95% CI (0.032, 0.211)]. These findings underscore the substantial role of cognitive reserve in improving overall and specific cognitive domains. Nonetheless, CRS’s influence was not statistically significant for other assessments, including MMSE, ADL, Hamilton Anxiety (HAMA), and Depression (HAMD) scales, after factoring in confounding variables. These findings underscore the importance of cognitive reserve in enhancing cognitive function, particularly in memory and executive domains, highlighting potential targets for cognitive intervention.

**Table 3:** Cognitive reserve and neuropsychological outcomes (*n* = 369).

Variable	Adjustment	*R*	B Coefficient (β)	Standard Error (SE)	*t*-value	*p*-value	95% Confidence Interval
CRS score impact on MMSE	Unadjusted	0.136	0.021	0.008	2.627	0.009	[0.005, 0.037]
	Adjusted	0.284	0.011	0.008	1.363	0.174	[−0.005, 0.027]
CRS score impact on MoCA	Unadjusted	0.186	0.050	0.014	3.625	<0.001	[0.023, 0.077]
	Adjusted	0.387	0.027	0.014	2.015	0.045	[0.001, 0.054]
CRS score impact on ANT	Unadjusted	0.148	0.072	0.025	2.868	0.004	[0.022, 0.121]
	Adjusted	0.311	0.042	0.025	1.661	0.098	[−0.008, 0.092]
CRS score impact on MES	Unadjusted	0.221	0.202	0.047	4.347	<0.001	[0.111, 0.294]
	Adjusted	0.425	0.121	0.045	2.672	0.008	[0.032, 0.211]
CRS score impact on ADL	Unadjusted	0.131	−0.015	0.006	−2.530	0.012	[−0.027, −0.003]
	Adjusted	0.291	−0.011	0.006	−1.848	0.065	[−0.023, 0.001]
CRS score impact on HAMA	Unadjusted	0.095	−0.034	0.019	−1.831	0.068	[−0.071, 0.003]
	Adjusted	0.127	−0.027	0.020	−1.384	0.167	[−0.065, 0.011]
CRS score impact on HAMD	Unadjusted	0.050	−0.015	0.016	−0.961	0.337	[−0.046, 0.016]
	Adjusted	0.110	−0.008	0.017	−0.458	0.647	[−0.040, 0.025]

The adjusted variables ware age, gender, and education level.

### 3.4 Association of cognitive reserve with age, education level, and occupation

The analysis underscores a significant association between higher CRS scores and younger age, higher education levels, and intellectual occupations. Detailed findings in Table 4 reveal that both education and occupation levels significantly correlate with CRS scores after adjusting for age (*p* < 0.001). However, when further adjusting for education, the association between occupation type and CRS diminishes, indicating no significant differences in cognitive reserve between individuals in intellectual versus physical occupations (*p* = 0.118). Conversely, higher education remains a strong predictor of higher CRS scores, even after adjusting for age and occupation (*p* < 0.001), highlighting the robust impact of educational attainment on cognitive reserve.

**Table 4:** Association of CRS with education level and occupation (*n* = 369).

Variable	*n*	CRS Score	Univariate *p*	Adjusted-1 *p*	Adjusted-2 *p*	Adjusted-3 *p*
Age group, %*			0.031	–	–	–
<65 years old	251	45.54 (12.17)				
> = 65 years old	118	40.65 (11.41)				
Education, %*			<0.001	<0.001	–	<0.001
Lower education	91	36.49 (10.96)				
Higher education	278	44.62 (11.65)				
Occupation, %*			<0.001	<0.001	0.118	–
Physical	76	37.38 (12.45)				
Intellectual	293	43.97 (11.51)				

*CRS Score as the dependent variable. The adjusted-1 p-values were education and occupation as independent variables, age as covariate. The adjusted-2 P-values were occupation as independent variables, age and education as covariate. The adjusted-3 p-values were education as independent variables, age, and occupation as the covariate.

Additionally, factorial ANOVA results presented in Table 5 illustrate the effects of education and occupation on CRS, with age as a covariate. The interaction between education and occupation does not significantly affect CRS (*p* = 0.529), further emphasizing the predominant influence of education level over occupational type in contributing to cognitive reserve.

**Table 5:** Factorial ANOVA for effects of education and occupation on CRS, with age as a covariate (*n* = 369).

Source of variation	DF	Sum of squares	Mean square	*F*-Value	*p*-Value	Effect size (η^2^)
Education	1	2049.255	2049.255	15.56	<0.001	0.041
Occupation	1	321.548	321.548	2.441	0.119	0.007
Education × Occupation	1	52.396	52.396	0.398	0.529	0.001
Age (Covariate)	1	80.425	80.425	0.611	0.435	0.002

DF, Degrees of freedom.

### 3.5 Association of cognitive impairment with education level and occupation categories

The study findings reveal a significant association between cognitive impairment and education-and-occupation combinations, with higher education and physical occupation [OR = 0.316 (0.153,0.654), *p* = 0.002] or intellectual occupation [OR = 0.211 (0.063,0.710), *p* = 0.012] displaying lower risks of cognitive impairment than low education and physical occupation combinations in Table 6. The results also indicate that occupation has a notable influence on scores on overall assessments of cognition (MMSE, *p* = 0.013 and MoCA *p* = 0.002) and some assessments of specific cognitive domains such as memory and language cognition domain (MES *p* < 0.001 and ANT *p* < 0.001). However, no significant effect of occupation was observed on scores on the ADL, HAMA or HAMD (*p* > 0.05). These findings are presented in Supplementary Table S1.

**Table 6:** Association of cognitive impairment with education level and occupation categories (*n* = 369).

Education/Occupation	Univariate OR (95% CI)	*p*-value	Model 1 OR (95% CI)	*p*-value	Model 2 OR (95% CI)	*p*-value	Model 3 OR (95% CI)	*p*-value
Higher/Physical (*n* = 255)	0.303 (0.151, 0.609)	0.001	0.347 (0.170, 0.710)	0.004	0.347 (0.170, 0.710)	0.004	0.316 (0.153, 0.654)	0.002
Lower/Intellectual (*n* = 53)	0.755 (0.326, 1.749)	0.512	0.743 (0.314, 1.757)	0.499	0.743 (0.314, 1.757)	0.499	0.719 (0.301, 1.717)	0.457
Higher/Intellectual (*n* = 23)	0.755 (0.326, 1.749)	0.008	0.228 (0.068, 0.758)	0.016	0.228 (0.068, 0.758)	0.016	0.211 (0.063, 0.710)	0.012

CI, Confidence Interval. Reference: lower education and physical occupation (*n* = 38). Model 1: Logistic regression model adjusted for age, sex, and ethnicity. Model 2: Logistic regression model adjusted for age, sex, ethnicity, and CRS score. Model 3: Logistic regression model adjusted for age, sex, ethnicity, CRS score, and brain reserve (head circumference).

## 4 Discussion

Our study underscores the significant role of educational attainment in reducing the risk of cognitive impairment, highlighting that higher education is associated with better cognitive activity and lower risk, irrespective of occupational complexity. Contrary to initial assumptions, intellectually demanding occupations did not correlate with reduced cognitive impairment risk or enhanced cognitive activity. Analysis of cognitive function assessments revealed that occupation impacts global cognition scores (MMSE, MoCA) and specific cognitive domains (MES, ANT), with intellectual occupations linked to superior verbal skills, delayed recall, and visuospatial abilities compared to physical occupations. Notably, individuals with higher education exhibited lower cognitive impairment risks across both physical and intellectual occupations, emphasizing education as a pivotal factor in cognitive health. These findings suggest that while occupation influences certain cognitive skills, education remains a more critical determinant of overall cognitive resilience.

### 4.1 Education is associated with cognitive impairment

We found a strong association between education level and cognitive impairment. Similar to the findings of a Brazilian study on the association between education level and cognition (*n* = 1,023), in the present study, education predicted better cognitive abilities even after adjusting for age and cognitive activity (Suemoto et al., 2022). Low education had the highest contribution to dementia risk in China among nine modifiable risk factors, with 10.8% of dementia cases attributable to low education (Mukadam et al., 2019). Moreover, research conducted in China has shown that low education is one of the highest contributing factors to the risk of dementia. In fact, a significant proportion of dementia cases in China have been attributed to low education levels. This is particularly noteworthy given that China has historically had a high illiteracy rate, with as much as 80% of the population being illiterate in 1949 (Wang et al., 2020). It is widely accepted that education level is considered an early contributor to the cognitive reserve previously shown to protect against cognitive impairment independent of the brain reserve. These findings underscore the importance of education in promoting cognitive health and reducing the risk of cognitive decline.

It is worth noting that previous research has suggested that the relationship between cognitive impairment and cognitive reserve may be modified by education level (Wang et al., 2020). Notably, a recent study conducted by the Religious Orders Study and Memory Aging Project (ROSMAP) involving 752 participants found that education level was only associated with baseline cognitive function, and was not related to slower rates of cognitive decline, later onset of decline, dementia onset or death, or residual cognitive decline not attributable to the neuropathologic burden. These findings contradict many of the tenets of cognitive reserve theory (Wilson et al., 2019).

### 4.2 Occupation type is not associated with cognitive impairment

The present study aimed to investigate the relationship between occupation type and cognitive function. While previous research has yielded mixed results on the matter, recent studies have shown that individuals with intellectual occupations tend to exhibit higher cognitive performance than those with physical occupations. The current research further explored this relationship and found that scores on cognitive domains such as language, delayed recall, and visuospatial/executive function were indeed higher in individuals with intellectual occupations.

It is worth noting, however, that the link between occupation and cognitive function is complex. While some studies suggest that occupational complexity protects against dementia (Dekhtyar et al., 2015; Ojagbemi et al., 2016), others have found no such association (Chapko et al., 2018). Occupational complexity is a downstream representative of cognitive reserve, which is directly influenced by educational attainment in childhood and early adulthood. The reduced risk of dementia in individuals with specific careers may be related to exposure to stressful work-related events, rather than solely to the effects of cognitively stimulating activities on cognitive reserve (Hülür et al., 2020).

In this study, we found that occupation was not related to cognitive reserve and cognitive impairment, and that intellectual occupations tended to be associated with higher levels of cognitive reserve through higher education. These findings suggest that individuals with intellectually stimulating occupations may have greater cognitive reserve, resulting in better cognitive performance in later life. Further exploration of the relationship between occupation and cognitive function is warranted.

The results of our study align with those of a previous study conducted in Cuba, the Dominican Republic, Venezuela, Peru, Mexico, and China (Prince et al., 2012), which found that education level, but not occupation, protected against dementia. However, another cross-sectional study in China (Ma et al., 2022) found that low occupational complexity explained a significant proportion of the variation in dementia cases, when both education level and occupation were considered. It is important to note that neither of these studies evaluated brain reserve. To our knowledge, our study is the first to investigate the association between education level and occupation, while also considering brain reserve information. Our study provides strong evidence for the role of education level as a cognitive reserve agent and for its ability to predict cognitive impairment. We examined data from 369 physical examination participants in a developing country (China) and administered a complete neuropsychological assessment. This association between socioeconomic status and cognitive function differs from previous studies examining cognitive reserve over the lifespan (Chapko et al., 2018).

## 5 Limitations and conclusions

However, the limitations of the study also need to be highlighted. This study was cross-sectional study; thus, participants were not followed over time. We obtained education level and occupation data across the lifespan and used multiple cognitive domain assessments to achieve a complete assessment of cognitive function, cognitive activity, and brain reserve. The CRS was also used to divide cognitive activity into multiple periods, providing a longitudinal perspective regarding the association between cognitive reserve and cognitive ability. Despite all precautions regarding the quality of clinical information provided by participants, recall bias was unavoidable; we reduced recall bias to some extent by training our interviewers to spot conflicting answers to questionnaires and seeking confirmation from family members about areas of doubt. Although selection bias was another threat to study validity, we contacted the regional medical supervisor, who confirmed that the demographics of our sample were similar to his perceptions of the local population, which suggests that our sample was representative of the local context. In addition, residual confounding persisted even after model adjustments for sociodemographic variables. Another area for improvement is the possibility of poor participant compliance and incorrect questionnaire responses due to the lengthy neuropsychological tests conducted in this study. We considered this issue and provided a refreshment room with coffee, food, and automatic massage chairs to alleviate fatigue. Finally, occupation significantly influenced some overall cognitive scores and individual cognitive domains but not rates of cognitive impairment, suggesting that larger sample sizes are needed to confirm our findings.

In conclusion, our study of 369 participants predominantly with higher education levels and physical occupations demonstrates that educational attainment significantly influences cognitive activity throughout life and is linked to reduced cognitive impairment risk. Conversely, occupational complexity does not appear to impact cognitive impairment or cognitive activity. Future longitudinal studies in developing countries with detailed lifestyle and cognitive activity measurements are essential to further elucidate the role of cognitive reserve in preventing cognitive impairment and dementia.

## Data availability statement

The raw data supporting the conclusions of this article will be made available by the authors, without undue reservation.

## Ethics statement

The studies involving humans were approved by the Ethics Committee of the School of Nursing, Jilin University. The studies were conducted in accordance with the local legislation and institutional requirements. The participants provided their written informed consent to participate in this study.

## Author contributions

TZ: Writing – original draft, Writing – review & editing, Data curation, Software. SL: Writing – original draft, Writing – review & editing, Data curation. PL: Data curation, Writing – original draft, Writing – review & editing. YW: Resources, Writing – original draft, Writing – review & editing. LC: Project administration, Supervision, Writing – original draft, Writing – review & editing.
