# Supplementary figures and images for: The impact of education and occupation on cognitive impairment: a cross-sectional study in China
Source: Front Aging Neurosci. 2024 Jul 11;16:1435626. doi: 10.3389/fnagi.2024.1435626 (PMC11273364; doi:10.3389/fnagi.2024.1435626)

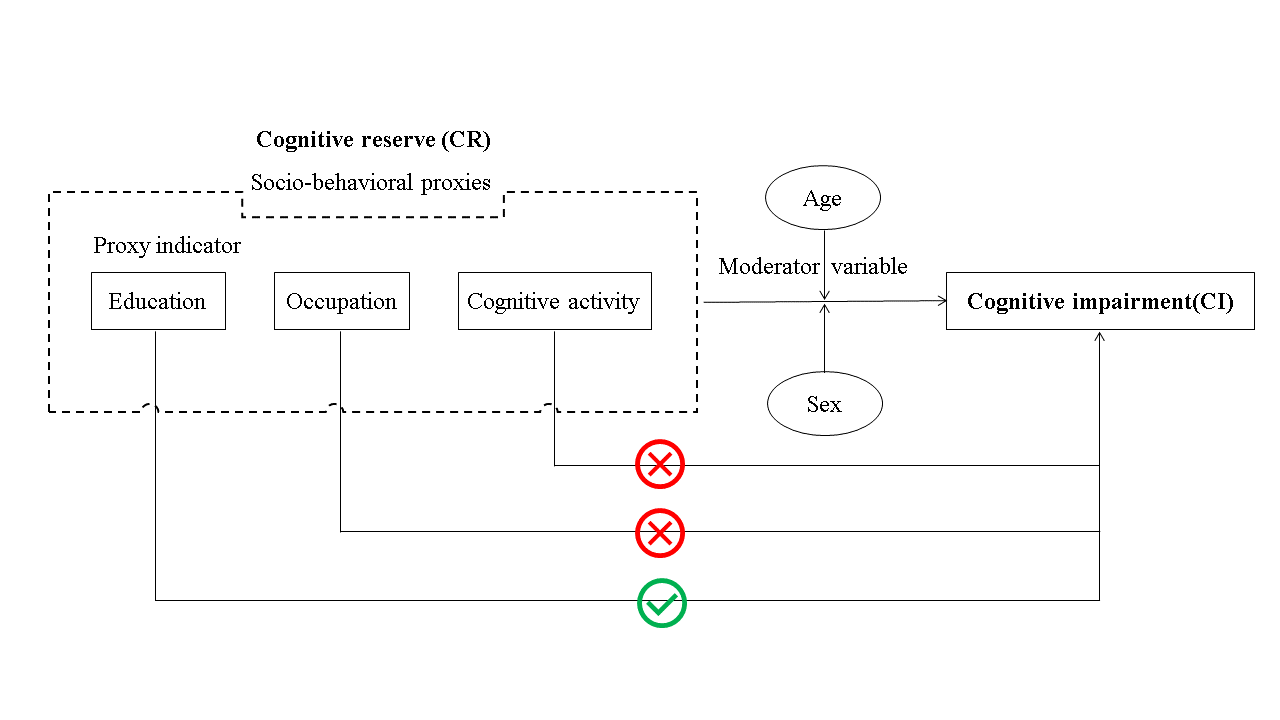

Supplement: Supplementary file 1 [file Image_1.PNG]
