# Supplementary material for: The impact of education and occupation on cognitive impairment: a cross-sectional study in China
Source: Front Aging Neurosci. 2024 Jul 11;16:1435626. doi: 10.3389/fnagi.2024.1435626 (PMC11273364; doi:10.3389/fnagi.2024.1435626)
Supplement: Supplementary file 2 [file Table_1.docx]

**Supplementary Tables**

Supplementary Table 1. Neuropsychological test scores according to occupation (n=369)

| **Scale** | **Physical occupation** | **Intellectual occupation** | ***p*** |
| --- | --- | --- | --- |
|  | **n=76** | **n=293** |  |
| **Total MMSE score, mean (SD)*** | 27.7 (2.9) | 28.5 (1.5) | 0.013 |
| Orientation | 9.6 (1.3) | 9.9 (0.4) | 0.134 |
| Memory | 3.0 (0.3) | 2.9 (0.3) | 0.767 |
| Attention and calculation | 4.3 (1.3) | 4.5 (0.9) | 0.114 |
| Delayed memory | 2.1 (0.9) | 2.3 (0.8) | 0.038 |
| Language | 7.8 (0.4) | 7.9 (0.4) | 0.385 |
| Visuospatial skills | 0.8 (0.4) | 0.9 (0.3) | 0.057 |
| **Total MoCA score, mean (SD)*** | 24.1 (4.4) | 25.9 (2.7) | 0.002 |
| Visuospatial/executive | 3.6 (1.5) | 3.8 (1.3) | 0.315 |
| Naming | 2.7 (0.6) | 2.9 (0.4) | 0.063 |
| Attention | 4.7 (1.7) | 5.1 (1.3) | 0.071 |
| Language | 2.5 (0.7) | 2.6 (0.7) | 0.499 |
| Abstraction | 1.7 (0.6) | 1.9 (0.4) | 0.011 |
| Delayed recall | 2.1 (1.4) | 2.8 (1.5) | <0.001 |
| Orientation | 5.8 (0.9) | 6.0 (0.1) | 0.089 |
| **Total ADL score, mean (SD)*** | 20.6 (2.0) | 20.3 (1.2) | 0.166 |
| BADL | 8.3 (0.7) | 8.2 (0.6) | 0.570 |
| IADL | 12.1 (1.1) | 12.1 (1.0) | 0.499 |
| **HAMD score, M (IQR)** ‡ | 3.0 (4.0) | 2.0 (5.0) | 0.311 |
| **HAMA score, M (IQR)** ‡ | 4.0 (5.0) | 3.0 (5.0) | 0.531 |
| **Total MES score, mean (SD) *** | 82.0 (14.1) | 88.1 (9.6) | <0.001 |
| Memory | 37.6 (9.9) | 42.0 (7.0) | <0.001 |
| Execution | 44.2 (8.3) | 46.1 (5.9) | 0.065 |
| **Total ANT score, mean (SD)*** | 17.8 (5.5) | 20.5 (5.8) | <0.001 |

*one-way ANOVA, ^†^chi-square test, ‡ Nonparametric tests
